# P03 Effects of a dietary supplement containing probiotics, cinnamon and cranberry extracts in the prevention of recurrent cystitis

**DOI:** 10.1093/jacamr/dlad077.006

**Published:** 2023-08-02

**Authors:** S Ait Abdellah, Q Dauchet, A Leblanc, J M Bohbot

**Affiliations:** PiLeJe Laboratoire, 31-35 rue de la Fédération, 75015 Paris, France; PiLeJe Laboratoire, 31-35 rue de la Fédération, 75015 Paris, France; PiLeJe Laboratoire, 31-35 rue de la Fédération, 75015 Paris, France; Institut Alfred Fournier, 25 Bd Saint Jacques, 75014 Paris, France

## Abstract

**Objectives:**

A study was conducted to evaluate the effects of a dietary supplement containing two probiotic strains (*Lactobacillus helveticus* LA201 and *Lactobacillus plantarum* BL3706, 5×10^9^ cfu per two tablets) and extracts of cinnamon (320 mg) and cranberry (100 mg) in the prevention of recurrent cystitis.

**Methods:**

A total of 80 women (18–65 years) were included who reported at least two episodes of cystitis in the past 6 months, at least one of which was a bacterial cystitis confirmed by a urine cytobacteriological examination or a physician, and for whom the episodes of cystitis had a strong impact on their quality of life (QoL; Acute Cystitis Symptom Score [ACSS] QoL questionnaire >2). Participants took two tablets of the supplement per day for 6 months (*n*=37; Group 1) or no supplement (*n*=38; Group 2). The ACSS questionnaire was completed every 15 days, or in case of cystitis, for 8 months (total score ranged from 0 [no urinary discomfort] to 39 [maximum discomfort]). The two groups were comparable at baseline, with a higher proportion of postmenopausal women in Group 1.

**Results:**

During the 6 months, 2/3 of the women in Group 1 had no recurrence of cystitis (0 symptomatic cystitis episodes [SCE]) while 2/3 of the women in Group 2 had at least one SCE (*P*=0.03 Fisher test). The total ACSS score during SCE was significantly lower in Group 1 (8.4±5.1 versus 12±5.9, *P*<0.0001 ANOVA), with results in favour of Group 1 for dimensions assessing typical symptoms of acute cystitis and QoL. Compliance and tolerance were excellent.

**Conclusions:**

These results suggest that the supplement may help prevent recurrence of cystitis, particularly in postmenopausal women who are at high risk.

